# Allergen Uptake, Activation, and IL-23 Production by Pulmonary Myeloid DCs Drives Airway Hyperresponsiveness in Asthma-Susceptible Mice

**DOI:** 10.1371/journal.pone.0003879

**Published:** 2008-12-08

**Authors:** Ian P. Lewkowich, Stephane Lajoie, Jennifer R. Clark, Nancy S. Herman, Alyssa A. Sproles, Marsha Wills-Karp

**Affiliations:** 1 Division of Immunobiology, Cincinnati Children's Hospital Medical Center, University of Cincinnati College of Medicine, Cincinnati, Ohio, United States of America; 2 Department of Pediatrics, University of Cincinnati College of Medicine, Cincinnati, Ohio, United States of America; New York University School of Medicine, United States of America

## Abstract

Maladaptive, Th2-polarized inflammatory responses are integral to the pathogenesis of allergic asthma. As regulators of T cell activation, dendritic cells (DCs) are important mediators of allergic asthma, yet the precise signals which render endogenous DCs “pro-asthmatic”, and the extent to which these signals are regulated by the pulmonary environment and host genetics, remains unclear. Comparative phenotypic and functional analysis of pulmonary DC populations in mice susceptible (A/J), or resistant (C3H) to experimental asthma, revealed that susceptibility to airway hyperresponsiveness is associated with preferential myeloid DC (mDC) allergen uptake, and production of Th17-skewing cytokines (IL-6, IL-23), whereas resistance is associated with increased allergen uptake by plasmacytoid DCs. Surprisingly, adoptive transfer of syngeneic HDM-pulsed bone marrow derived mDCs (BMDCs) to the lungs of C3H mice markedly enhanced lung IL-17A production, and rendered them susceptible to allergen-driven airway hyperresponsiveness. Characterization of these BMDCs revealed levels of antigen uptake, and Th17 promoting cytokine production similar to that observed in pulmonary mDCs from susceptible A/J mice. Collectively these data demonstrate that the lung environment present in asthma-resistant mice promotes robust pDC allergen uptake, activation, and limits Th17-skewing cytokine production responsible for driving pathologic T cell responses central to the development of allergen-induced airway hyperresponsiveness.

## Introduction

Allergic asthma, a disease that continues to rise in both incidence and morbidity in the developed world, manifests as recurrent episodes of wheezing, shortness of breath and coughing in response to environmental stimuli. Although the origins of asthma are complex, it is accepted that asthma results from an inappropriate, Th2-dominated immune response to environmental allergens in genetically predisposed individuals. In asthmatic individuals, allergen exposure facilitates expansion of pathogenic, allergen-specific Th2 cells producing IL-4, IL-5 and IL-13, cytokines which induce pulmonary eosinophilic inflammation, IgE synthesis, airway wall remodeling and airway hyperresponsiveness (AHR), hallmarks of allergic asthma [1]. In contrast, non-asthmatic individuals respond in a fundamentally different way, becoming tolerized through the activation of immunosuppressive CD4+CD25+ regulatory T cells secreting IL-10 and TGFβ [2], [3]. However, despite an increasingly sophisticated understanding of the pathogenesis of allergic asthma, the factors that promote the initial development of a pathogenic versus protective T cell response remain unknown.

As professional antigen presenting cells, dendritic cells (DCs) are capable of skewing T cell differentiation towards a pathogenic or regulatory phenotype through a number of mechanisms. For example, the panel of co-stimulatory molecules expressed by DCs plays a role in the type of T cell response elicited - CD86 and OX40L contribute to the development of pathogenic Th2 cells, [4]–[6] while ICOS-L and PD-1/PD-L promote the development of protective regulatory T cells [7]–[11]. DCs also directly regulate T cell differentiation by producing cytokines that drive the differentiation/expansion of Th2 cells (IL-6 alone [12], [13]), Th17 cells (IL-6+TGFβ/IL-23 [12], [14], [15]), Tregs (TGFβ/IL-10 [16]–[18]) or Th1 cells (IL-12 [19]). Identification of a DC-produced cytokine directly responsible for inducing the development of Th2 cells remains elusive, yet the Th2-skewing capacity of DCs can be influenced by a number of factors [20]–[25]. Finally, the recent identification of distinct DC subsets has provided another mechanism through which DCs may control the development of pathogenic or regulatory T cell responses. Myeloid DCs (mDCs), are effective T cell stimulators, inducing a variety of effector T cell responses (e.g. Th1, Th2 or Th17) dependent upon the types of stimuli they receive, and the compartment from which they are isolated [26]–[28]. In contrast, while plasmacytoid DCs (pDCs) strongly induce the development of regulatory T cells as a result of elevated PD-L1 expression [29]–[31], they can also induce the development of IFNγ-producing Th1 cells after viral exposure, or Th2 differentiation after exposure to IL-3 [32], [33]. Thus, DCs can adapt to conditions present at the time of antigen encounter, promoting responses appropriate to the specific antigen, a response further fine-tuned by the types of DC subset involved in antigen presentation.

Dendritic cells in the lung play an important role in the development of allergic airway responses. In the large airways, DCs form a network below the epithelial cell layer [34]–[36], a position from which they sample and transport inhaled antigens present in the airway lumen to lung-draining LNs, where T cell activation occurs [37]. Airway DCs increase in numbers following allergen challenge in both human, and animal models [38], [39] suggesting an important role for these cells in asthma. Indeed, transfer of Ag-pulsed, bone marrow-derived mDCs to the airways of naïve animals is sufficient to sensitize mice for subsequent development of allergic asthma [40]–[43]. In contrast, adoptive transfer of Ag-pulsed, bone marrow-derived pDCs induces tolerance [29], elegantly demonstrating that *in vitro* derived DC subsets have markedly differing capacities to induce pathogenic or protective T cell responses. However, *in vivo*, the precise subset of DCs responsible for inducing pathogenic versus protective T cell responses, the pattern of DC-derived cytokines that drive these T cell responses, and the extent to which DC subset recruitment and cytokine production are regulated by the pulmonary environment and genetics in asthma-susceptible and resistant hosts, remains unclear.

To address these questions, we utilized a murine model of susceptibility to allergen-induced airway hyperresponsiveness (AHR) wherein intratracheal administration of house dust mite (HDM) induces the development of AHR in susceptible A/J animals, but has little impact on AHR in resistant C3H mice. Using this model, we demonstrate that following allergen exposure in susceptible hosts, allergen uptake is predominantly by pulmonary mDCs capable of high level co-stimulatory molecule expression and the production of Th17-promoting cytokines production (IL-6, IL-23), and that these factors are critical for the development of allergen-induced airway hyperresponsiveness. Moreover, transfer of mDCs with a susceptible phenotype completely reconstitutes susceptibility in resistant hosts. Taken together, our results suggest that factors in the pulmonary environment regulate the activation of mDCs, controlling the expression of high levels of co-stimulatory molecules, and Th17-promoting cytokines (IL-6, IL-23) critical for the development of T-cell dependent airway hyperresponsiveness.

## Results

### Development of HDM-based model of experimental asthma

**Figure 1:**
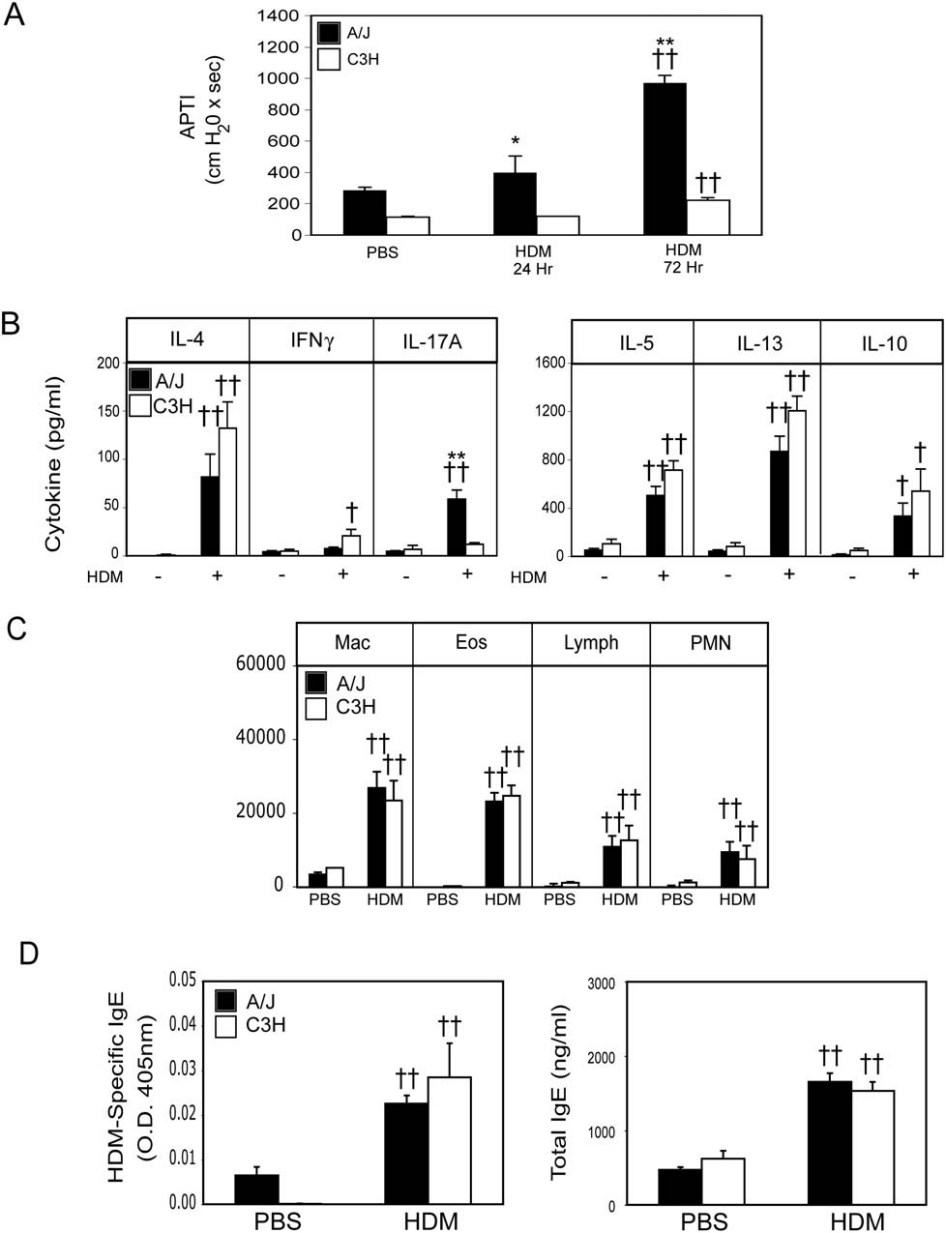
Intratracheal HDM exposure induces AHR and a mixed Th2/Th17 cytokine profile in A/J, but not C3H mice. A/J (solid bars) and C3H (open bars) mice were sensitized and challenged with PBS or HDM as described in Materials and Methods. Mice were sacrificed 24 or 72 hours after the second HDM/PBS exposure and AHR was assessed (A). Cytokine levels were determined in lung cell cultures stimulated with medium alone, or in the presence of HDM (30 µg/ml) (B). Cellular composition of the BAL fluid was determined (C). Serum total and HDM-specific IgE levels were quantified (D). Mean+SEM shown. N = 12 mice from 3 independent experiments. * and ** indicate significant differences of p<0.05 and p<0.001 respectively between A/J and C3H animals. † and †† indicate significant differences compared to PBS-treated animals, p<0.05 and p<0.001 respectively.

To test the hypothesis that differences in susceptibility to allergen-driven AHR in A/J and C3H mice result from differential regulation of pulmonary DC recruitment or activity, we developed a model in which allergen sensitization occurs via the airways. Specifically, we examined the impact of i.t. administration of house dust mite extract (HDM), a natural aeroallergen, on subsequent development of allergen-induced AHR. As shown in Figure 1a, 24 hours after a second i.t. HDM exposure, AHR rose in A/J animals, reaching a plateau 4–5 fold higher than baseline by 72 hours. In contrast, in C3H mice, airway responses remained indistinguishable from baseline at 24 hours, and were only modestly increased (<2-fold) 72 hours after allergen exposure. Interestingly, in spite of the well-documented role of IL-13 as a central mediator of allergic asthma [44], production of Th2 cytokines (IL-4, IL-5, IL-13) was similar in HDM re-stimulated lung cell cultures from A/J and C3H mice, while production of IFNγ was induced only in lung cell cultures from C3H mice (Figure 1b). However, the levels of IFNγ are quite low, indicating a clear Th2 bias to immune response in both resistant and susceptible animals and suggesting that resistance is not associated with the preferential induction of a Th1 response in this model. Interestingly, lung cell cultures from susceptible A/J mice produced significantly greater amounts of IL-17A upon HDM-restimulation, suggesting that concomitant induction of Th2 and Th17 responses may be required for the manifestation of AHR in allergen-sensitized hosts (Figure 1b). HDM exposure also induced a similar degree of BAL eosinophilia (Figure 1c), and total and HDM-specific IgE synthesis (Figure 1d) in A/J and C3H mice. Interestingly, despite the elevated IL-17A production observed in HDM-restimulated lung cell cultures from A/J mice, the degree of BAL neutrophilia was indistinguishable in A/J and C3H mice (Figure 1c). It is important to note that, while the C3H mice used in these studies possess a mutation in TLR4 rendering them resistant to LPS signaling, the inability to respond to LPS does not seem to underlie their genetic resistance, as C3H mice which do not possess the mutation in TLR4 (C3H/HeN) respond identically to HDM (data not shown). Thus, to assess the importance of endogenous DC subsets in controlling resistance or susceptibility in a genetic model of asthma, we made use of the HDM-based model of experimental asthma in subsequent studies.

### Allergen uptake by pulmonary DC subsets in resistant and susceptible mice

To assess whether genetic differences responsible for conferring susceptibility to the development of allergen-induced AHR are associated with changes in the balance of mDCs and pDCs taking up allergen at the time of sensitization, A/J and C3H mice were given a single i.t. dose of PBS or AlexaFluor 405 labeled-HDM (AF405-HDM), sacrificed 24 or 72 hours later, and lung DC subsets were quantified by flow cytometry. We classified 7-AAD^neg^CD11b^+^CD11c^+^Gr1^neg^ cells as mDCs while pDCs were classified as 7-AAD^neg^CD11b^neg^CD11c^+^Gr1^+^ (Figure S1). To confirm that the cells we identified were, in fact, mDCs and pDCs, we further analysed gated DC population for expression of B220, and CD317, markers of pDCs [45], [46]. While pDCs expressed CD317 at the highest levels, as measured by surface staining with mPDCA-1 and 120g8, neutrophils also expressed this marker (Figure S1). Indeed, CD317 was recently found to be expressed not only on pDCs, but also upregulated upon cells following activation with type I or type II IFNs [46], suggesting that a portion of neutrophils in the lung may be activated following HDM exposure. In contrast, only pDCs expressed B220 to any extent (Figure S1). Collectively, these data suggest that the 7-AAD^neg^CD11b^neg^CD11c^+^Gr1^+^ in these studies are pDCs.

**Figure 2:**
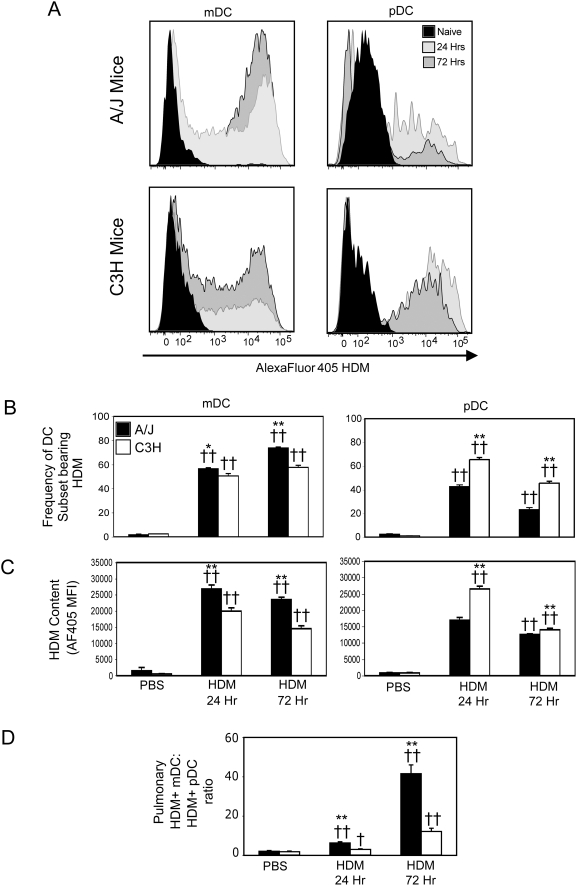
Susceptibility to allergen-induced AHR is associated with increased allergen uptake by pulmonary mDCs. Mice were sensitized with PBS or AF405-HDM as described in Materials and Methods. AF405 staining of gated mDCs (left panels) and pDCs (right panels) from naïve mice (solid black histograms) and mice 24 hours (light grey histograms) or 72 hours (dark grey histograms) after allergen exposure was determined by flow cytometry (A). Proportion of total mDCs (left panels) and pDCs (right panels) containing allergen in A/J (solid bars) and C3H (open bars) mice (B) and allergen content (C) was determined by flow cytometry. Ratio of HDM+ mDCs to HDM+ pDCs was calculated (D). Mean+SEM shown (n = groups of 8 mice in 2 independent experiments). * and ** indicate significant differences of p<0.05 and p<0.001 respectively between A/J and C3H animals. †† indicates significant differences compared to PBS-treated animals, p<0.001.

Gating on mDCs or pDCs from AF405-HDM-treated A/J and C3H mice revealed that both mDCs and pDCs had taken up allergen (Figure 2a). However, compared to C3H mice at similar time points, in A/J mice, we observed a significantly greater proportion of mDCs that contained allergen (Figure 2b). On a per cell basis, HDM^+^ mDCs from A/J mice also contained significantly greater levels of allergen (as measured by AF405-HDM MFI) compared to mDCs from C3H mice (Figure 2c). In contrast, examination of pDCs revealed that at both 24 and 72 hours after allergen challenge, those from C3H mice were more frequently positive for allergen (Figure 2b), and contained more allergen per cell (Figure 2c), than those from A/J animals. As a direct measure of the balance of immunogenic mDCs and tolerogenic pDCs in the lung, we also determined the ratio of HDM^+^ mDCs to HDM^+^ pDCs in the lung at various times after challenge. As shown in Figure 2d, in both A/J and C3H mice, the mDC:pDC ratio increased steadily over time after allergen challenge, demonstrating that allergen uptake by immunogenic mDCs accompanies AHR development (Figure 2d). However, the HDM^+^ mDC:HDM^+^ pDC ratio was significantly elevated in the lungs of A/J mice (41±4 at 72 hours), compared to C3H mice (12±1 at 72 hours). Collectively, this data suggests that development of airway responses is associated with a markedly increased frequency of allergen bearing pulmonary mDCs and a concomitantly decreased frequency of allergen-bearing pulmonary pDCs.

As pulmonary DCs have also been demonstrated to play an important role in promoting asthma exacerbations in previously sensitized animals [47], we also wished to determine if the balance of allergen-presenting DCs was similarly skewed towards mDCs in genetically susceptible mice that had been previously sensitized. Mice sacrificed 24 and 72 hours after a second allergen exposure to AF405-HDM displayed a similar bias in allergen uptake by pulmonary DC subsets – in A/J mice, allergen uptake by mDCs predominated, while pDCs from resistant C3H mice were frequently allergen positive (Figure S2). Thus, in susceptible mice, allergen uptake is principally by immunogenic mDCs, while in resistant mice, the balance of allergen uptake is shifted towards pDCs.

### Co-stimulatory molecule expression on pulmonary DC subsets

**Figure 3:**
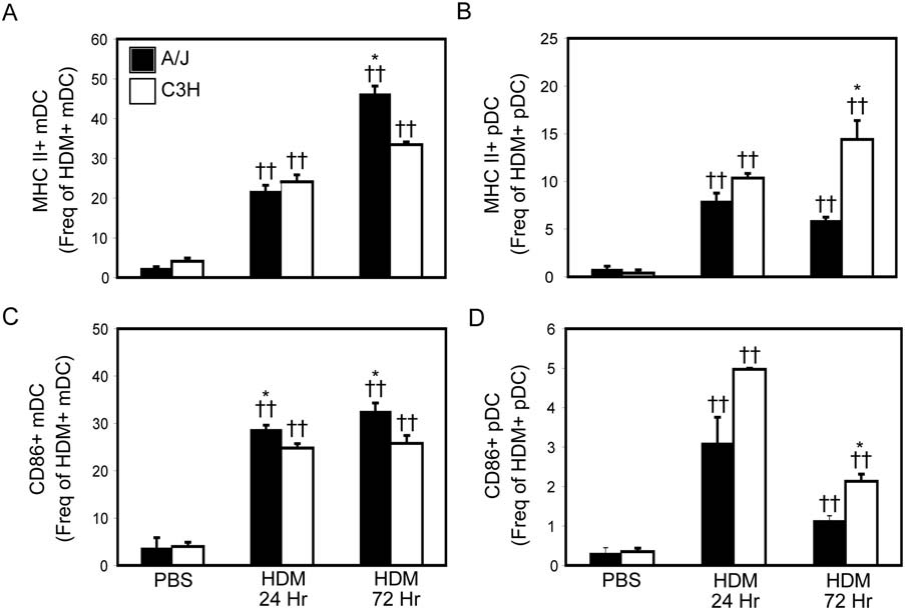
Significantly enhanced co-stimulatory molecule expression on HDM-bearing mDCs from A/J mice. A/J (solid bars) and C3H (open bars) mice were treated with PBS or AF405-labeled HDM as described in Materials and Methods. Mice were sacrificed 24 and 72 hours after the second HDM/PBS exposure and lungs were removed. Proportion of HDM^+^ mDCs (A) or pDCs (B) expressing MHC Class II, and the proportion of HDM^+^ mDCs (C) or pDCs (D) expression CD86, was determined by flow cytometry. Mean+SEM shown. n = 8 mice from 2 experiments. * indicates significant differences of p<0.05 between A/J and C3H animals. †† indicate significant differences of p<0.001 compared to PBS-treated animals.

As expression of molecules involved in antigen presentation and T cell co-stimulation are required for activation of naïve T cells, we also compared the expression of MHC Class II and CD86 on HDM^+^ pulmonary mDCs from susceptible and resistant mice 24 and 72 hours after sensitization with AF405-HDM. Interestingly, we observed that a greater frequency of HDM+ mDCs from A/J mice stained positive for CD86 and MHC Class II at 24 and 72 hours after allergen challenge by flow cytometry, compared to those from C3H mice (Fig 3a & c). Moreover, HDM exposure also increased the number of CD86 and MHC Class II molecules (as indicated by MFI) on the surface of mDCs from A/J mice compared to those from C3H mice (Table 1). Similar results were observed with CD80, OX40L and ICOS-L (data not shown). Examining co-stimulatory molecule expression on pDCs revealed that the proportion of HDM^+^ pDCs expressing CD86 or MHC Class II was consistently lower (∼2 fold) in susceptible A/J mice compared to C3H mice, (Figures 3b & 3d). Moreover, HDM exposure induced significantly higher levels of MHC Class II on HDM^+^ pDCs from C3H mice than those from A/J mice (Table 1). Interestingly, pDCs generally expressed lower levels of MHC Class II than mDCs from the same mouse (Table 1), supporting previous reports suggesting that, compared to mDCs, pDCs have limited T cell stimulatory activity. Collectively, these data demonstrate that the development of AHR in genetically susceptible animals is associated with enhanced activation and function of pulmonary mDCs following allergen sensitization. In contrast, in resistant animals, there is a shift towards increased activation and allergen uptake by pulmonary pDCs.

**Table 1:** Co-stimulatory molecule expression on HDM pulmonary DC populations following allergen exposure of A/J and C3H mice.

Treatment	CD86 MFI mDC		MHC MFI mDC	
	A/J	C3H	A/J	C3H
PBS Treated	2,215±496	1,528±37	6,066±2,066	7,192±2,537
HDM - 24 Hours	3,315±360 *	2,325±92	27,748±3,947 *	16,070±713
HDM - 72 Hours	2,473±170 *	1,967±110	6,797±806	6,760±557

Treatment	CD86 MFI pDC		MHC MFI pDC	
	A/J	C3H	A/J	C3H
PBS Treated	1,699±1,698	1,545±1,500	6,236±1,807	6,420±1,993
HDM - 24 Hours	3,368±145	4,042±680	7,418±843 *	11,877±991
HDM - 72 Hours	3,006±107	2,854±101	6,273±474	5,340±86

a - mDC defined as CD11c^+^, CD11b^+^, Gr1^−^

b - pDC defined as CD11c^+^, CD11b^−^, Gr1^+^

* - indicates significant differences of p<0.05 between strains

### Differences in allergen uptake are not due to differential DC subset recruitment

**Figure 4:**
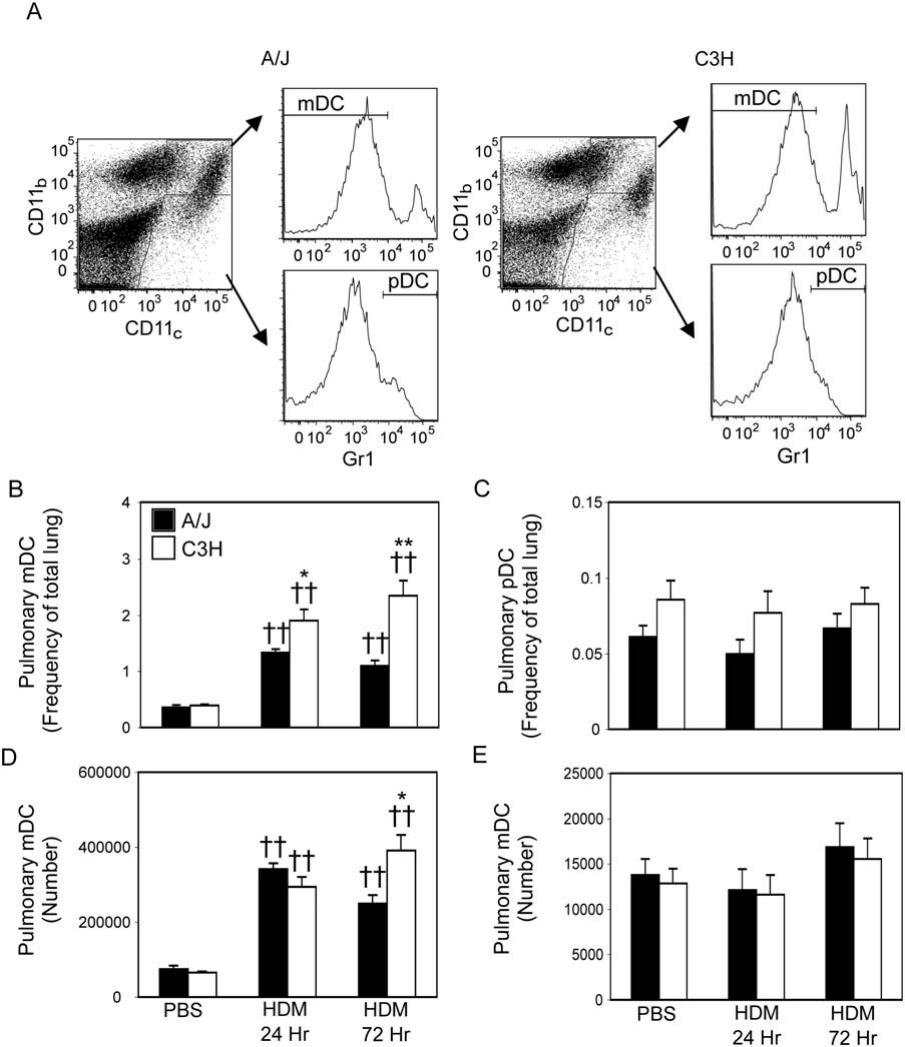
DCs or both subsets are more numerous in the lungs of resistant animals. A/J (solid bars) and C3H (open bars) mice were sensitized with PBS or HDM as described in Materials and Methods. Mice were sacrificed 24 and 72 hours after the second HDM/PBS exposure and lungs were removed. mDCs and pDCs were identified by flow cytometry (A). The frequency (B) and absolute (C) number of mDCs and the frequency (D) and absolute numbers (E) of pDCs in the lungs of PBS- or HDM-treated animals was determined. Mean+SEM shown. n = 8 mice from 2 independent experiments. * or ** indicates significant differences of p<0.05 or p<0.001 respectively between A/J and C3H animals. †† indicates a significant difference of p<0.001 compared to PBS-treated animals.

To determine if increased allergen uptake and activation of mDCs (A/J mice) or pDCs (C3H mice) observed following intratracheal sensitization resulted from a greater overall recruitment of the dominant DC subset to the lung, we examined the number of total mDCs and pDCs in the lungs of A/J and C3H mice at 24 and 72 hours after HDM sensitization. As shown in Figure 4a, HDM exposure significantly increased mDC recruitment to the lungs of A/J and C3H mice (compare to Figure S1). However, at both 24 and 72 hours after HDM exposure, the proportion (Figure 4b), and number (Figure 4d) of lung cells classified as mDCs was significantly greater in C3H mice compared to similarly treated A/J mice. In contrast, HDM exposure had little impact on pDC recruitment in either strain and pDCs comprised a similar percentage of total lung cells in both A/J and C3H mice (Figure 4c). While HDM exposure had little impact on absolute pDC number at 24 hours after allergen exposure, it tended to increase the number of pDCs present in the lung after 72 hours, although this did not reach statistical significance (Figure 4e). Thus, while mDCs are strongly recruited to the lungs following allergen exposure, they are present, overall, in lower numbers in A/J mice compared to C3H mice, suggesting that the comparatively increased allergen uptake observed by A/J mDCs is not simply a reflection of more mDCs available for allergen uptake.

### Similarly skewed allergen uptake by DC subsets is observed in lung-draining LNs

**Figure 5:**
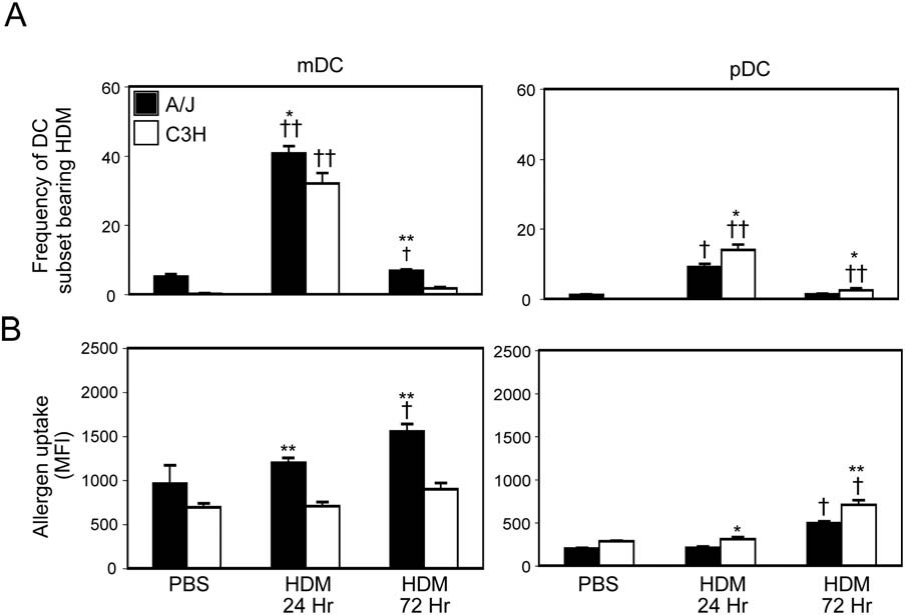
Susceptibility to allergen-induced AHR is associated with increased allergen uptake by lung-draining LN mDCs. A/J (solid bars) and C3H (open bars) mice were treated with PBS or AF405-labeled HDM as described in Materials and Methods. Mice were sacrificed 24 and 72 hours after the second HDM/PBS exposure and lung-draining LNs were removed. (A) Frequency of HDM+ mDCs, and (B) mean AF405 intensity was determined in gated mDCs (left panels) and pDCs (right panels) from A/J and C3H mice. Mean+SEM shown (n = 8 mice from 2 independent experiments). * and ** indicate significant differences between A/J and C3H animals (p<0.05 and p<0.001 respectively). † and †† indicate significant differences compared to PBS-treated animals, p<0.05 and p<0.001.

As initial T cell activation occurs in the lung-draining LN after allergen sensitization, we compared the distribution of allergen in DC subsets in the lung-draining LNs of HDM-AF405 sensitized A/J and C3H mice. We find that recruitment of HDM^+^ DC subsets to the LN peaked at 24 hours, remaining slightly elevated at 72 hours in both strains (Figure 5a). In contrast, the AF405-HDM MFI in mDCs and pDCs continued to rise, peaking at 72 hours in both strains (Figure 5b). Comparison of allergen uptake in DC subsets revealed that compared to C3H mice, more mDCs from A/J mice contained allergen, while in C3H mice, a significantly greater proportion of pDCs contained allergen (Figure 5a). In comparing AF405-HDM MFI, we observed that, compared to those from resistant mice, mDCs from susceptible animals contained more allergen at all time points, while pDCs from resistant mice contained significantly greater levels of allergen than those from A/J animals (Figure 5b). Thus, endogenous DC subsets in the lungs and the lung-draining LNs display similar biases in allergen uptake, suggesting that the selective accumulation of either HDM^+^ mDCs in susceptible mice, or HDM^+^ pDCs in resistant mice may promote the development of a pathogenic or regulatory T cell response, respectively.

### In vivo HDM exposure reduces Th17-associated cytokine production in resistant mice

**Figure 6:**
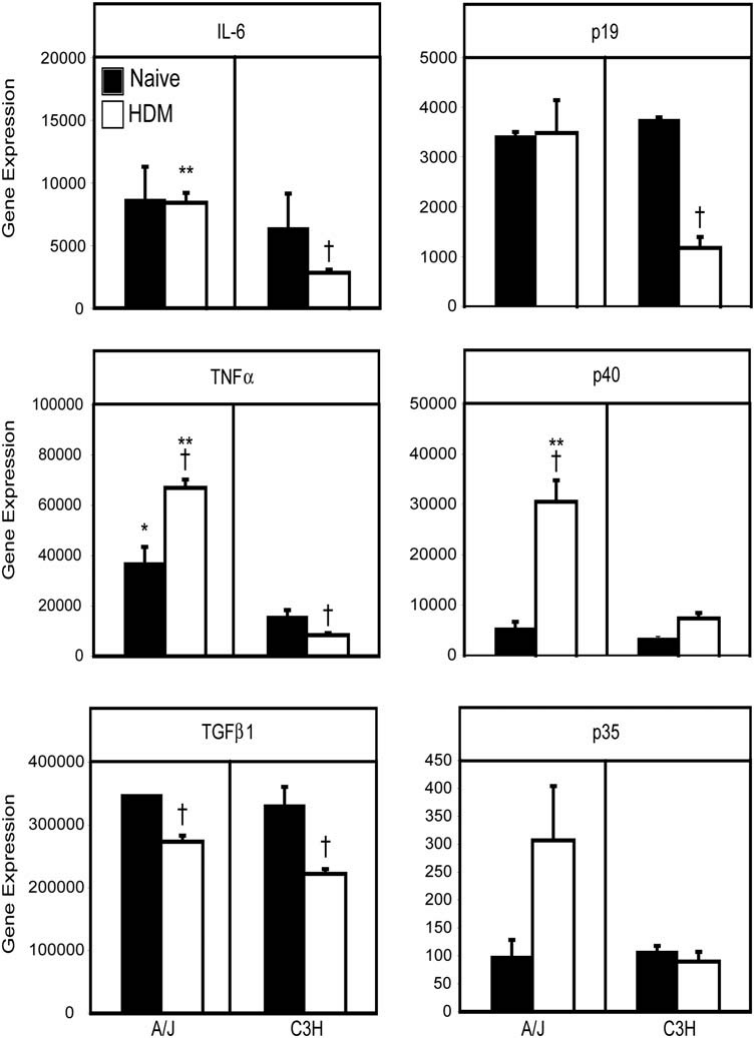
HDM exposure increases cytokine expression in pulmonary mDCs from susceptible, but not resistant mice. A/J and C3H mice were sensitized with a single dose of HDM as described in Materials and Methods. Mice were sacrificed before allergen challenge, or 48 hours after a single allergen exposure challenge, pulmonary mDCs were isolated by FACS sorting, and total RNA was isolated. Expression of IL-6, IL-23 p19, TNFα, IL-12/23 p40, TGFβ1 and IL-12 p35 were determined by real time PCR. Representative data from 1 of 3 experiments shown. * and ** indicate significant differences between A/J and C3H mice, p<0.05 and p<0.001 respectively . † indicates significant differences compared to naïve mice, p<0.05.

As the nature of cytokines produced by DCs directs T cell differentiation, we also compared pulmonary mDC and pDC expression of the T cell skewing cytokines IL-6, TNFα, TGFβ1, and p40, a component of both IL-12 (when paired with p35) and IL-23 (when paired with p19) in DCs from both strains of mice. We harvested total RNA from flow-sorted, pulmonary mDCs and pDCs from naïve, and HDM-exposed A/J and C3H mice 48 hours following a single allergen exposure for real time RT-PCR analysis of cytokine expression. As shown in Figure 6, mDCs from naïve lungs of both strains contained high, but comparable, levels of message for TNFα, TGFβ1, and IL-6, and slightly lower (but similar) levels of mRNA for p19 and p40 (Figure 6). In contrast, mDCs from both strains contained very little message for p35 (Figure 6), resulting in a high p19:p35 ratio, suggesting that pulmonary mDCs may be biased towards IL-23 production, potentially supporting the expansion of Th17 cells. Interestingly, the impact of HDM exposure on mDC cytokine mRNA levels was different in the two strains. In C3H mice, HDM reduced pulmonary mDC cytokine production – IL-6, p19, TNFα, and TGFβ1 mRNA were significantly decreased in mDCs from HDM-treated C3H mice compared to naïve controls (Figure 6). In contrast, levels of cytokine message were significantly increased (TNFα, p40), or present at comparable levels (p19, IL-6) in mDCs from HDM-sensitized A/J mice compared to mDCs from naïve A/J mice (Figure 6). Together, these results suggest that the response of pulmonary mDCs to inhaled allergen differs in resistant and susceptible hosts. Moreover, as HDM exposure reduces p19, IL-6 and TNFα production in resistant, but not susceptible mice, this may explain the limited production of IL-17A observed in resistant animals (Figure 1).

As it is still unclear how pDCs promote protective responses to inhaled allergen, we also examined the impact of HDM on the expression of cytokines by pulmonary pDCs. Comparing the cytokine profile of mDCs and pDCs, we found that both subsets make a similar pattern of cytokines, although pDCs expressed them at lower levels (Figure S3). One notable difference between mDCs and pDCs however – while the p19:p35 mRNA ratio was elevated in mDCs, p19 and p35 were expressed at similar levels in pulmonary pDCs, suggesting that pDCs may produce more IL-12 than mDCs (Figure S3). Comparison of cytokine message in naïve pDCs from A/J and C3H mice revealed comparable levels of IL-6, TNFα, TGFβ1. While levels of p40 and p19 tended to be lower in pDCs from naïve C3H mice compared to those from A/J mice, this did not reach statistical significance (Figure S3). Following HDM exposure however, pDCs from A/J mice were stimulated to express higher levels of message for IL-6 and TNFα (Figure S3), but cytokine production by C3H pDCs was unaffected by HDM exposure. Collectively, these data suggest that aeroallergen-stimulated cytokine production by pulmonary mDCs and pDCs differs in genetically resistant and susceptible animals - HDM exposure does not significantly inhibit cytokine production by pulmonary DCs in susceptible animals, while such exposure decreases DC cytokine production in resistant animals.

### Similar HDM uptake and co-stimulatory molecule expression on bone marrow-derived mDCs from A/J and C3H mice

**Figure 7:**
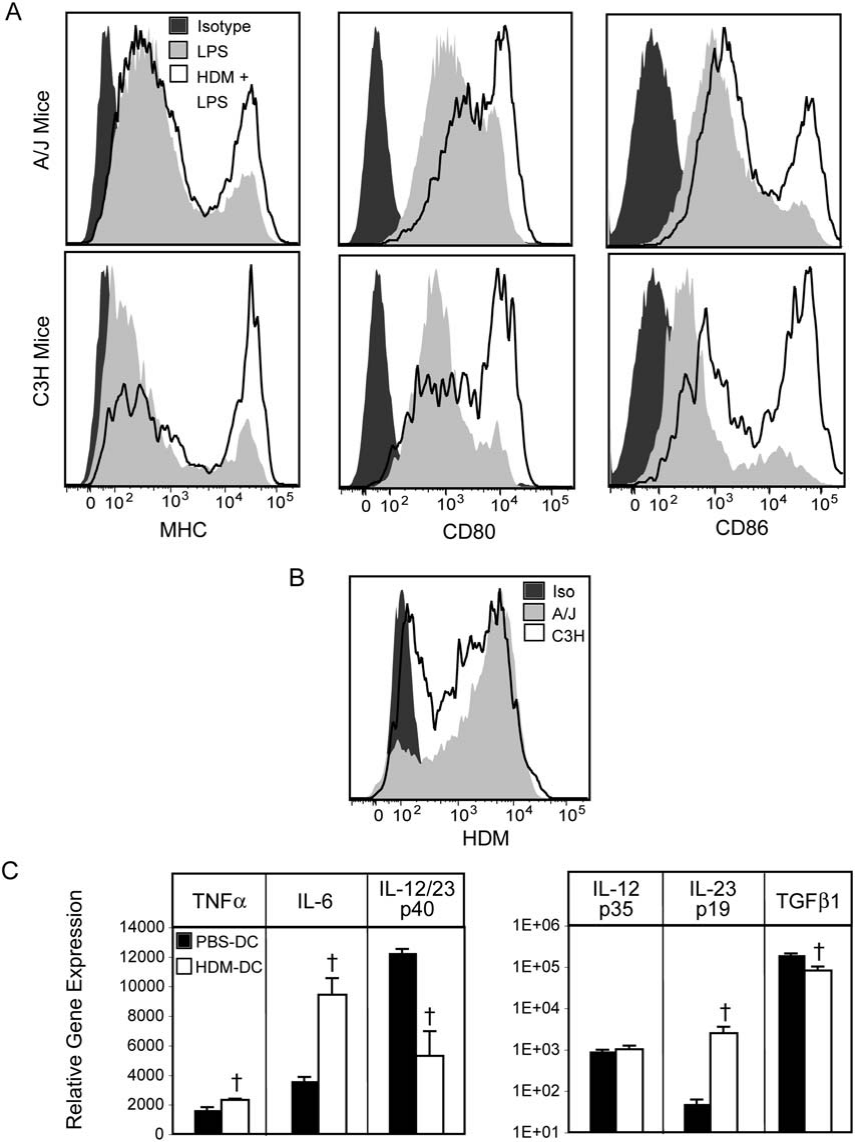
*In vitro* allergen exposure of BMDCs from resistant mice increases cytokine production and co-stimulatory molecule expression. mDCs were derived by culturing bone marrow cells in the presence of GM-CSF and IL-4 for 6 days. On day 7, BMDC were pulsed with 30 µg/ml of HDM or medium. On day 8, BMDCs were matured by the addition of 1 µg/ml LPS. (A) After overnight culture, expression of the co-stimulatory molecules MHC, CD80 and CD86 on LPS (light grey histograms) or LPS+HDM (open histograms) pulsed BMDCs was determined by flow cytometry. Isotype staining is indicated by dark grey histogram. (B) BMDC from A/J (light grey histogram) or C3H (open histogram) mice were cultured with HDM-AF405 for 48 hours, and allergen uptake was assessed by flow cytometry. Cells pulsed with unlabelled HDM shown (solid black histogram). Representative plots from 1 of 2 experiments shown. (C) Cytokine expression by bone marrow-derived mDCs used to sensitize mice was determined by real time PCR analysis, Mean+SEM shown (n = 8 BMDC samples from 2 independent experiments). † indicates significant differences compared to PBS-pulsed DCs, p<0.05.

To determine if the differing responses of endogenous A/J and C3H mDCs to HDM was a result of an intrinsic difference in mDC function, or the environment in which the DCs matured (i.e. the allergen-exposed lung), we exposed bone marrow-derived dendritic cells (BMDCs) from these two strains to HDM *in vitro*. Bone marrow cells cultured with GM-CSF and IL-4 for 6 days [conditions that induce the development of mDCs [48]] were pulsed with PBS or AF405-HDM for 24 hours, matured overnight with LPS, and co-stimulatory molecule expression, antigen uptake and cytokine expression were assayed. Compared to LPS-matured BMDCs from A/J mice, those from C3H mice expressed slightly less MHC Class II and CD86 and substantially less CD80 (Figure 7a), likely the result of impaired LPS-induced maturation resulting from the lack of functional TLR4 in C3H mice [49]. Surprisingly, in contrast to the limited impact of HDM exposure on co-stimulatory molecule expression by C3H mDCs *in vivo*, *in vitro* HDM-pulsed, LPS-matured C3H BMDCs displayed similar (or slightly elevated) induction of co-stimulatory molecule expression, compared to similarly treated BMDCs from A/J animals (Figure 7a) suggesting that differences in HDM-induced mDC maturation between strains is not due to differential responsiveness to LPS. Moreover, in contrast to the low levels of allergen captured by C3H mDCs following *in vivo* AF405-HDM exposure, when BMDC allergen uptake was compared, a similar capacity for allergen uptake was observed in BMDCs from A/J and C3H mice (Figure 7b). Interestingly, in contrast to the inhibitory impact of *in vivo* HDM exposure on production of IL-6, TNFα and p19 by mDCs from C3H mice, mDCs from C3H mice cultured with HDM *in vitro* up-regulate the expression of these cytokines (Figure 7c). Strikingly, this pattern of HDM-induced cytokine production is similar to that observed in mDCs from the lungs of sensitized A/J animals (Figure 6), suggesting that over-expression of these cytokines by pulmonary mDCs may be an important susceptibility mediator in A/J mice. While LPS-matured BMDCs from A/J animals expressed much higher levels of cytokine mRNA than those from C3H mice (likely due to the LPS-unresponsiveness of BMDCs from C3H mice), pulsing A/J BMDCs with HDM induced a pattern of cytokine expression associated with promoting/maintaining Th17 responses (elevated IL-6, TGF-β, IL-23 p19 and comparatively less IL-12 p35) (Figure S4). Moreover, the levels of cytokine expression induced following HDM exposure of BMDCs from A/J mice was comparable to the levels observed in similarly treated BMDCs from resistant C3H mice (Figure S4). Collectively, these results suggest that during maturation in the lung, mDCs from resistant mice are exposed to factors that limit allergen uptake, co-stimulatory molecule expression and cytokine synthesis normally observed following *in vivo* HDM exposure.

### Adoptive transfer of bone marrow-derived mDCs renders genetically resistant mice susceptible to the development of AHR

**Figure 8:**
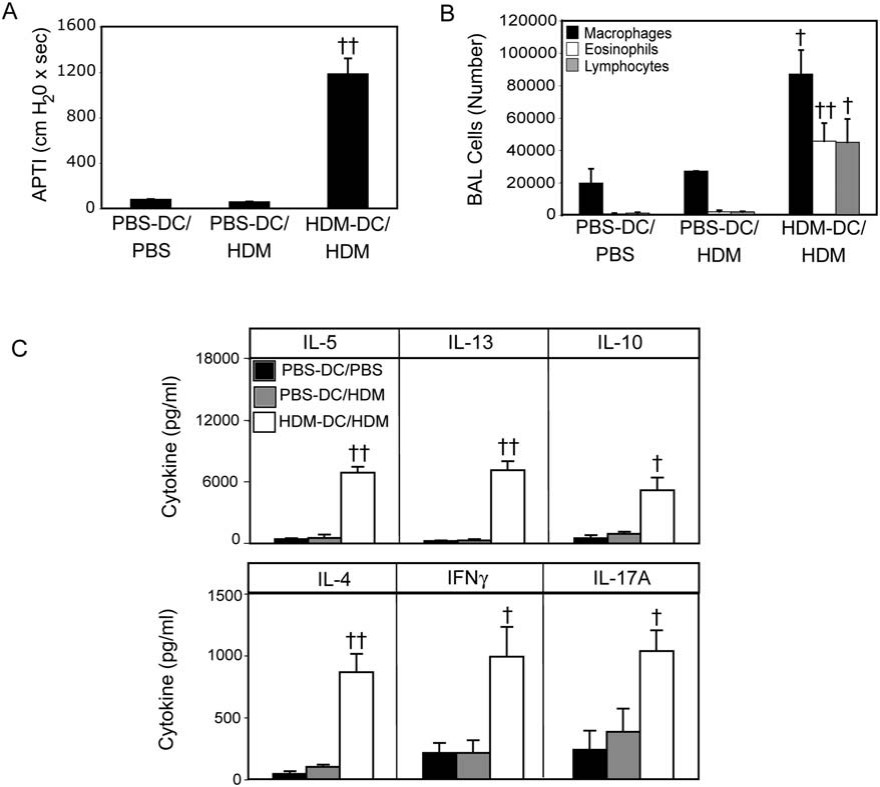
Adoptive transfer of mDCs to resistant mice induces AHR and IL-17 production similar to that seen in susceptible animals. C3H mice were sensitized with PBS-, or HDM-pulsed bone marrow-derived DCs on day 0. Mice were challenged with HDM or PBS on day 14 and sacrificed 72 hours later. AHR (A) and BAL cell composition (B) were examined at sacrifice. Lung cells were also stimulated with HDM *in vitro*, and cytokine production was assayed by ELISA (C). † and †† indicate significant differences (p<0.05 and p<0.001 respectively) compared to PBS-treated animals or PBS-pulsed DCs.

To determine if shifting the phenotype of mDCs involved in allergen presentation in resistant mice towards one of robust allergen uptake, high-level co-stimulatory molecule expression, and production of Th17-associated cytokines is sufficient to cause AHR in genetically resistant animals, we adoptively transferred 1×10^6^ *in vitro* PBS- or HDM-pulsed C3H BMDCs to the airways of C3H mice on day 0, and challenged with PBS or HDM on day 14. Mice were sacrificed on day 17 to assess airway function, and pulmonary T cell cytokine expression. In C3H mice sensitized with PBS-pulsed dendritic cells, subsequent challenge with PBS or HDM, resulted in levels of AHR (Figure 8a), airway eosinophilia (Figure 8b), and HDM-restimulated Th2 cytokine production (Figure 8c) comparable to that seen in PBS-sensitized and challenged animals (compare to Figure 1a). In contrast, adoptive transfer of HDM-pulsed BMDCs induced robust AHR (Figure 8a), and airway eosinophilia (Figure 8b). Strikingly, the level of AHR induced in resistant C3H mice following sensitization with HDM-pulsed BMDCs was indistinguishable from that seen in HDM-exposed A/J mice (compare to Figure 1a). Lung cells from HDM-pulsed BMDC-sensitized C3H mice produced both Th2 (IL-4, IL-5, IL-13) and Th1 (IFNγ) cytokines (Figure 8c), similar to the pattern observed following sensitization with HDM, albeit ∼10-fold more intense (compare to Figure 1d). Interestingly, upon HDM-restimulation, lung cells from these BMDC-sensitized C3H mice produced levels of IL-17A 100-fold greater than those observed following intratracheal administration of HDM alone (Figure 8c). The concomitant induction of a Th17 response, similar to that observed in A/J mice treated with intratracheal HDM, is likely the result of elevated p19 and IL-6 production observed in adoptively transferred DCs (Figure 7c). Collectively, these results demonstrate that genetic resistance to allergen-induced AHR can be overcome by limiting the ability of pDCs to capture antigen and shifting the balance of antigen presentation towards highly activated, mDCs producing abundant Th17-skewing cytokines. Furthermore, these data also suggest that the pulmonary environment in which allergen encounter first takes place is of critical importance in directing the development of tolerance or allergen-induced AHR following initial allergen exposure.

## Discussion

Previous studies examining the roles of DC subsets in controlling allergic airway responses demonstrated a pro-asthmatic role for *in vitro-*derived mDCs, and a protective role for *in vitro-*derived pDCs [27], [29], [43], [50], yet were unable to determine how natural variations in endogenous DC subset activity influence the development of pathologic or protective T cell responses, what subset-specific factors control the development of these T cell responses *in vivo*, or what regulates production of these factors. Herein, we address these questions, demonstrating that while both mDCs and pDCs take up allergen in susceptible A/J mice, susceptibility to AHR is associated with preferential allergen uptake and increased co-stimulatory molecule expression by pulmonary mDCs. In contrast, in genetically resistant hosts, a greater frequency of pulmonary pDCs capture, and are activated by inhaled HDM. In addition, our studies provide several lines of evidence suggesting that susceptibility to allergen-induced AHR is associated with the capacity of mDCs to produce Th17-driving cytokines. First, pulmonary mDCs from genetically susceptible, allergen challenged mice produce high levels of IL-6 and IL-23, cytokines known to drive Th17 responses [14], [15], [51]. Secondly, in contrast to the limited IL-6 and IL-23 production by pulmonary mDCs from *in vivo* allergen-challenged C3H mice, BMDCs from C3H mice exposed to allergen *in vitro* produce high levels of IL-6 and IL-23, and adoptive transfer of these BMDCs induces the development of AHR indistinguishable from that seen in genetically susceptible mice. Collectively, our results suggest that in genetically susceptible animals, the pulmonary environment promotes allergen uptake and production of Th17-skewing cytokines by pulmonary mDCs that, in turn, promotes the development of pathologic T cell responses central to disease pathology.

Classically, Th2 cytokines are thought to be the principal effector molecules driving the development of experimental asthma [44], [52]. Surprisingly, our results suggest that susceptibility to allergen-driven AHR is associated with a mixed Th2/Th17 cytokine profile in the lung as both susceptible and resistant mice produced equivalent Th2 cytokines, but only susceptible mice produced IL-17A following HDM exposure. Furthermore, while similar patterns of Th1 and Th2 cytokine production were observed in C3H mice undergoing weak or robust airway responses, robust airway responses induced by adoptive transfer of HDM-pulsed BMDCs in normally resistant C3H mice were accompanied by a dramatic upregulation of IL-17A synthesis. The involvement of a mixed Th2/Th17 cytokine profile in asthma pathogenesis is supported by human studies showing increased production of IL-17A and infiltration of Th17 cells in the lungs of asthmatics, particularly those with more severe disease [53]–[56]. Although little is known about the specific role IL-17 plays in asthma, a recent report demonstrated that IL-17R-/- mice displayed reduced IgE production, airway eosinophilia and Th2 cytokine production, suggesting that IL-17A was required for allergic sensitization [57]. However, in the same study, neutralization of IL-17A during the effector phase enhanced AHR, suggesting a protective role in later stages of disease [57]. As we observe decreased production of Th17-skewing cytokines during the initiation of the response in genetically resistant animals, we speculate that Th17 cells play a pathogenic role in our model.

The observation of a mixed Th2/Th17 response in susceptible mice is in contrast to our previous observation of a clearly Th2-dominant pattern of cytokines in susceptible A/J mice and a preponderance of IFNγ in C3H mice in an OVA-based model [58]. Moreover, OVA-induced AHR in A/J mice was dependent upon IL-13 [44], and inhibited by IL-12 treatment [59], whereas in C3H mice, resistance was ablated by blockade of endogenous IL-12 [60], suggesting that the ability of natural allergens to efficiently induce pathogenic T cell responses may relate to their capacity to drive a mixed Th2/Th17 cytokine profile. The specific components of HDM that enhance its allergenicity remain unknown. However, while OVA is a single protein, HDM represents a complex mixture of components – both protein and non-protein. Two of these proteins, Der p1 and Der p2, possess potent proteolytic activity [61], [62], with the capacity to activate PAR-2 [63], and regulate of IgE production by cleaving the low affinity IgE receptor [64]. All of these biologic activities may potentially contribute to the allergenicity of this mixture. Another significant component of the HDM extract used in these studies is contaminating LPS. While low levels of LPS enhance the development of Th2 responses in the lung [65], and LPS responsiveness is limited in C3H/HeJ mice used in this study, we do not think that the failure of the C3H mice to respond to LPS is responsible for their lack of AHR in our model. While C3H mice are LPS-resistant, *in vitro* exposure to HDM+LPS induces similar levels of co-stimulatory molecule expression and allergen uptake in BMDCs from A/J and C3H mice. Moreover, we find that C3H/HeN mice, which possess a functional TLR4, respond to HDM identically to C3H/HeJ mice. Taken together these results suggest that any defects in allergen uptake or co-stimulatory molecule induction resulting from LPS unresponsiveness are overcome in the presence of HDM.

Interestingly, while *in vivo* HDM exposure inhibits allergen uptake, activation, and production of Th17-skewing cytokines by pulmonary mDCs from C3H mice, these parameters are maintained, or enhanced following exposure of bone marrow derived mDCs from resistant mice to HDM *in vitro*. Moreover, while pulmonary mDCs from resistant mice fail to induce AHR, transfer of bone marrow-derived mDCs pulsed with HDM *in vitro* induces robust AHR, demonstrating that DCs from different compartments possess different functional characteristics. This is wholly compatible with previous reports demonstrating that the type of signals the DC receive during maturation, and the tissues from which they are derived strongly influences their function [32], [33], [66]. Although the precise signals provided by the lung environment in C3H mice that limit pulmonary mDC activation are unclear, there are several possibilities. First, it is possible that some factor(s) differentially produced in the lungs of resistant and susceptible animals regulate the ability of pulmonary mDCs to respond to HDM. Interestingly, in the data reported here, pro-asthmatic BMDCs from C3H mice were differentiated in the presence of GM-CSF. Supporting a role for GM-CSF in enhancing the pro-asthmatic capacity of DCs, this cytokine selectively promotes the expansion of myeloid DCs *in vivo* [67], increases the expression of MHC Class II and CD86 on DCs in a murine model of asthma [24], and enhances the capacity of DCs to support Th2 differentiation in both mice [27], [67] and humans [68]. Interestingly, GM-CSF also enhances the production of IL-6 and IL-23 from DCs [69], leading us to hypothesize that the reduced capacity for co-stimulatory molecule expression and Th17-skewing cytokine production observed in C3H pulmonary mDCs may be the result of limited production of, or responsiveness to, GM-CSF *in vivo*. Studies are currently underway to test this hypothesis.

Our observation that shifting the balance of antigen presentation from activated pulmonary pDCs to activated mDCs is sufficient to induce airway responses in genetically resistant hosts also suggests that the presence of activated pDCs may limit the pro-asthmatic capacity of pulmonary mDCs. Such pDC-mediated inhibition of AHR may occur in a number of ways. We have previously demonstrated that in an mDC:T cell co-culture, pDCs significantly inhibit mDC-mediated T cell activation, suggesting that pDCs can directly limit mDC activity [70]. Additionally, while our *ex vivo* analysis of cytokine production by pulmonary pDCs did not reveal striking differences in cytokine production in pDCs between strains, it suggested that pDCs may be a source of IL-12, a cytokine that prevents allergen-induced AHR in this model [60]. Alternatively, as pDCs have been shown to be potent inducers of regulatory T cells [29]–[31], the increased activation of pulmonary pDCs observed in allergen-challenged resistant mice may contribute to a regulatory response responsible for controlling AHR. Consistent with this possibility, we previously demonstrated that *in vivo* Treg depletion in C3H mice significantly increased pulmonary mDC activity and AHR, while having no impact on these parameters in A/J mice [71]. Thus, through a variety of mechanisms, activated pDCs in the lungs of resistant animals may play a role in suppressing pulmonary mDC activation, and development of AHR.

The genetic factors underlying differential recruitment, capacity for allergen uptake, and Th17-driving cytokine production by pulmonary mDCs in susceptible and resistant mice remain unclear. However, we have previously identified two regions on murine chromosome 2 (*Abhr1* and *Abhr2*) that are strongly linked with asthma susceptibility in this model [58]. While mDCs from allergen-challenged A/J mice demonstrated enhanced capacity for allergen uptake (compared to those from C3H mice), fewer mDCs were present in the lungs of A/J animals, suggesting that genetic differences associated with susceptibility to allergen-induced AHR govern the function of distinct DC subsets, and not their selective recruitment. Close examination of the factors differentially expressed by pulmonary mDCs from A/J and C3H mice (IL-6, either IL-23 subunit, TNFα, co-stimulatory molecules) reveals that none are located within the genomic regions associated with susceptibility. However, C5a [absent in A/J mice due to a naturally occurring 2-base pair deletion [72]], located within *Abhr1* and strongly associated with the development of allergic asthma [73], has been shown to regulate IL-23 production [74] and the pulmonary mDC:pDC ratio [70]. Thus, in the lungs of resistant mice, production of complement components, likely by the epithelium, may negatively regulate mDC phagocytic capacity, activation, and production of Th17-driving cytokines, such that upon allergen exposure, the actions of pDCs predominate, and allow the development of an appropriate regulatory response.

Thus, our studies provide new insights into the phenotype of endogenous DCs that drive airway hyperresponsiveness. We demonstrate that genetic susceptibility to the development of allergen-induced AHR is the result of a pulmonary environment which, following allergen exposure, allows allergen presentation by mDCs expressing high levels of co-stimulatory molecules, and producing a Th17-promoting cytokine profile (IL-23 and IL-6). On the other hand, factors present in the lungs of resistant mice shift the balance of allergen uptake and activation from immunogenic mDCs to tolerogenic pDCs. Identification of the exact lung factors that preferentially regulate myeloid DC activation and promote the subsequent development of Th2/Th17 responses to harmless inhaled antigens should inform the development of improved therapies for the treatment of this ever increasing disease.

## Materials and Methods

### Mice

Male A/J and C3H/HeJ mice (5–6 weeks old, Jackson Laboratories, Bar Harbor, ME) were housed in a specific pathogen-free facility at Cincinnati Children's Hospital (Cincinnati, OH). Cincinnati Children's Hospital IACUC approved all animal protocols.

### Treatment protocols

Mice were treated with HDM extract (100 µg; Greer Laboratories, Lenoir, NC) or 40 µl PBS (Invitrogen, Carlsbad, CA) intratracheally (i.t.) on days 0 and 14. LPS content of the HDM extract was reported by the manufacturer to range between 0.043–0.047 EU/µg of protein. Mice were sacrificed 24 or 72 hours after HDM exposure for assessment of airway function and DC recruitment. Where indicated, AlexaFluor405 (Invitrogen, Carlsbad, CA) labeled HDM (AF405-HDM) was used. Alternatively, mice were sensitized with 2×10^6^ HDM-pulsed, bone marrow-derived mDCs.

### Development of mature, HDM-pulsed bone marrow derived myeloid DCs

Bone marrow cells (3×10^5^ cells/ml) were cultured in complete RPMI supplemented with GM-CSF (10 ng/ml, Peprotech, Rocky Hill, NJ, USA) and IL-4 (10 ng/ml, Peprotech). Medium was changed on day 3. On day 6, cells were cultured with HDM or AF405-HDM (30 µg/ml) and GM-CSF (10 ng/ml) for 24 hours. LPS was added at a final concentration of 1 µg/ml. 12 hours later the DCs were harvested, washed extensively in PBS and used for subsequent studies. BMDCs were consistently ∼95% mDCs, and expressed high levels of MHC Class II, CD80 and CD86.

### Assessment of allergen induced allergic responses

To evaluate airway responses, mice were anaesthetized, intubated and respirated at a rate of 120 breaths per minute with a constant tidal volume (0.2 ml) and paralyzed with decamethonium bromide (25 mg/kg) 72 hours after final allergen challenge. Acetylcholine (50 mg/kg) was injected into the inferior vena cava and dynamic airway pressure (cm H_2_0 x sec) was followed for 5 minutes. Immediately after AHR measurements, blood was collected to test for total and HDM specific IgE using Abs from Pharmingen (San Diego, CA). To collect BALF, lungs were lavaged three times with a 1.0-ml aliquot of cold Hanks' balanced salt solution (Invitrogen, Carlsbad, CA). Recovered lavage fluid (70 to 80%) was centrifuged (300*g* for 8 min) and the cell pellet resuspended in 1.0 ml of 10% FBS in PBS. Total cells were counted with a hemocytometer. Slides were prepared by cytocentrifugation (Cytospin 3; Shandon Instruments, Pittsburgh, PA), and stained with Diff-Quik (Dade Behring, Düdingen, Switzerland). Bronchoalveolar lavage (BAL) cell differential counts were determined using morphologic criteria under a light microscope with evaluation of ≥ 500 cells/slide.

### Lung and LN cell isolation

After AHR measurements, LNs and lungs were removed, minced and placed in 6 ml of RPMI 1640 containing Liberase CI (0.5 mg/ml)(Roche Diagnostics, Indianapolis, IN) and DNase I (0.5 mg/ml)(Sigma, St. Louis, MO) at 37°C for 45 minutes. The tissue was forced through a 70-micron cell strainer, and red blood cells were lysed with ACK lysis buffer (Invitrogen). Cells were washed with RPMI containing 10% FBS, viable cells were counted via trypan blue exclusion. Where indicated lung cells were cultured at 350,000 cells per well in a 96 well plate (250 µl final volume) with HDM (30 µg/ml). Tissue culture supernatants were harvested at 72 hours.

### Flow Cytometry

Staining reactions were performed at 4°C following incubation with FcBlock (mAb 2.4G2) for 30 minutes. Myeloid DCs (CD11c^+^, CD11b^+^, Gr1^−^) and plasmacytoid DCs (CD11c^+^, CD11b^−^, Gr1^+^) were quantified using anti-CD11c-APC (HL3), anti-CD11b-PE-Cy7 (M1/70), and anti-Gr1-APC-Cy7 (RB6-8C5). Co-stimulatory molecule expression was examined using PE-conjugated mAbs to MHC Class II (14-4-4s), CD86 (GL1), B7-H1 (MIH5) and B7-DC (TY25). Dead cells were excluded using 7-AAD. Data were acquired with an LSRII flow cytometer (BD Biosciences, San Jose, CA) equipped with lasers tuned to 488nm, 633nm, and 405nm. Cell sorts were performed on a FACSVantage SE flow cytometer (BD Biosciences) equipped with lasers tuned to 488 nm argon laser, and a 633 nm. Spectral overlap was compensated using the FACSDiVa software (BD Biosciences) and analyzed using FlowJo software (Treestar Inc., Ashland, OR).

### Determination of Cytokine Concentration

Cytokine levels in samples were measured by ELISA using matched antibody pairs purchased from Pharmingen (IL-4, IL-5) or R&D Systems (IL-13, Minneapolis, MN). Tissue culture supernatants were frozen at −80°C and thawed immediately prior to use.

### Quantitative real-time RT-PCR

To measure IL-12/23 p40, IL-23 p19, IL-6, IL-12 p35, TGFβ1 and TNFα message expression in sort-purified pulmonary mDCs or BMDCs, we used quantitative RT-PCR [75]. PCR primer pairs for IL-6 and IL-12/23 p40 were designed using Beacon Designer software, while primer pairs for IL-23/23p19 were obtained from the mouse primer depot [76]. Primers were: IL-6 sense ACA ACC ACG GCC TTC CCT AC; IL-6 anti-sense – AGC CTC CGA CTT GTG AAG TGG; IL-12/23 p40 sense – CAG AAG CTA ACC ATC TCC TGG; IL-12/23 p40 antisense – AGT CCA GTC CAC CTC TAC AAC; IL-23 p19 sense – GAC CCA CAA GGA CTC AAG GA; IL-23 p19 anti-sense – CAT GGG GCT ATC AGG GAG TA, IL-12 p35 sense – GCT TCT CCC ACA GGA GGT TT; IL-12 p35 anti-sense – CTA GAC AAG GGC ATG CTG GT; TNFα sense – CAT CTT CTC AAA ATT CGA GTG ACA A; TNFα anti-sense – TGG GAG TAG ACA AGG TAC AAC CC; TGFβ1 sense – AAT TCC TGG CGT TAC CTT GG; TGFβ1 anti-sense – GGC TGA TCC CGT TGA TTT CC. All primers were designed to span an intronic region to avoid co-amplification of genomic DNA.

### Statistical Analysis

To determine differences between multiple groups, analysis of variance (ANOVA) was used with *post hoc* comparisons using Fisher's method. For comparison between two groups, a Student's t-test was performed. Significance was assumed at p<0.05.

## Supporting Information

Figure S1 DC subsets found in the lung of mice. A/J mice treated with PBS were sacrificed, and lung cells stained with 7-AAD, and antibodies to CD11c, Gr1, and CD11b. 7-AAD- cells were gated for analysis of CD11b, CD11c, and Gr1 expression. (A) mDCs (CD11c+, CD11b+, Gr1−) and pDCs (CD11c+, CD11b−, Gr1+) cells can clearly be found in the lungs. Other major populations identified included neutrophils (CD11c+, CD11b+, Gr1+) and alveolar macrophages (CD11c+, CD11b−, Gr1−). (B) Gated DC population were analyzed for expression of B220, and CD317 with independent CD317-specific clones (120g8 and mPDCA-1).
(2.49 MB TIF)

Figure S2 Enhanced allergen uptake by pulmonary mDCs in susceptible mice is observed in previously sensitized animals. A/J (solid bars) and C3H (open bars) mice were sensitized and subsequently challenged with PBS or AF405-HDM as described in Materials and Methods. Proportion of total mDCs (left panels) and pDCs (right panels) containing allergen (A) and allergen content (B) was determined by flow cytometry. Mean+SEM shown (n = groups of 8 mice in 2 independent experiments). * and ** indicate significant differences of p<0.05 and p<0.001 respectively between A/J and C3H animals. †† indicates significant differences compared to PBS-treated animals, p<0.001.
(0.33 MB TIF)

Figure S3 Cytokine expression in pDCs from naïve and HDM-treated mice. A/J and C3H mice were sensitized with a single dose of HDM as described in Materials and Methods. Mice were sacrificed before allergen challenge, or 48 hours after a single allergen exposure challenge, pulmonary pDCs were isolated by FACS sorting, and RNA was isolated. Expression of IL-6, IL-23 p19, TNFα, IL-12/23 p40, TGFβ1 and IL-12 p35 were determined by real time PCR. Representative data from 1 of 3 experiments shown. * indicates significant differences between A/J and C3H mice, p<0.05
(0.45 MB TIF)

Figure S4 Cytokine expression by HDM-treated BMDCs from A/J mice. mDCs were derived by culturing bone marrow cells in the presence of GM-CSF and IL-4 for 6 days. On day 7, BMDC were pulsed with 30 µg/ml of HDM or medium. On day 8, BMDCs were matured by the addition of 1 µg/ml LPS. Cytokine expression by bone marrow-derived mDCs was determined by real time PCR analysis. Mean+SEM shown (n = 8 BMDC samples from 2 independent experiments). †† indicates significant differences compared to PBS-pulsed DCs, p<0.001.
(0.20 MB TIF)
