# Supplementary figures and images for: Allergen Uptake, Activation, and IL-23 Production by Pulmonary Myeloid DCs Drives Airway Hyperresponsiveness in Asthma-Susceptible Mice
Source: PLoS One. 2008 Dec 8;3(12):e3879. doi: 10.1371/journal.pone.0003879 (PMC2586658; doi:10.1371/journal.pone.0003879)

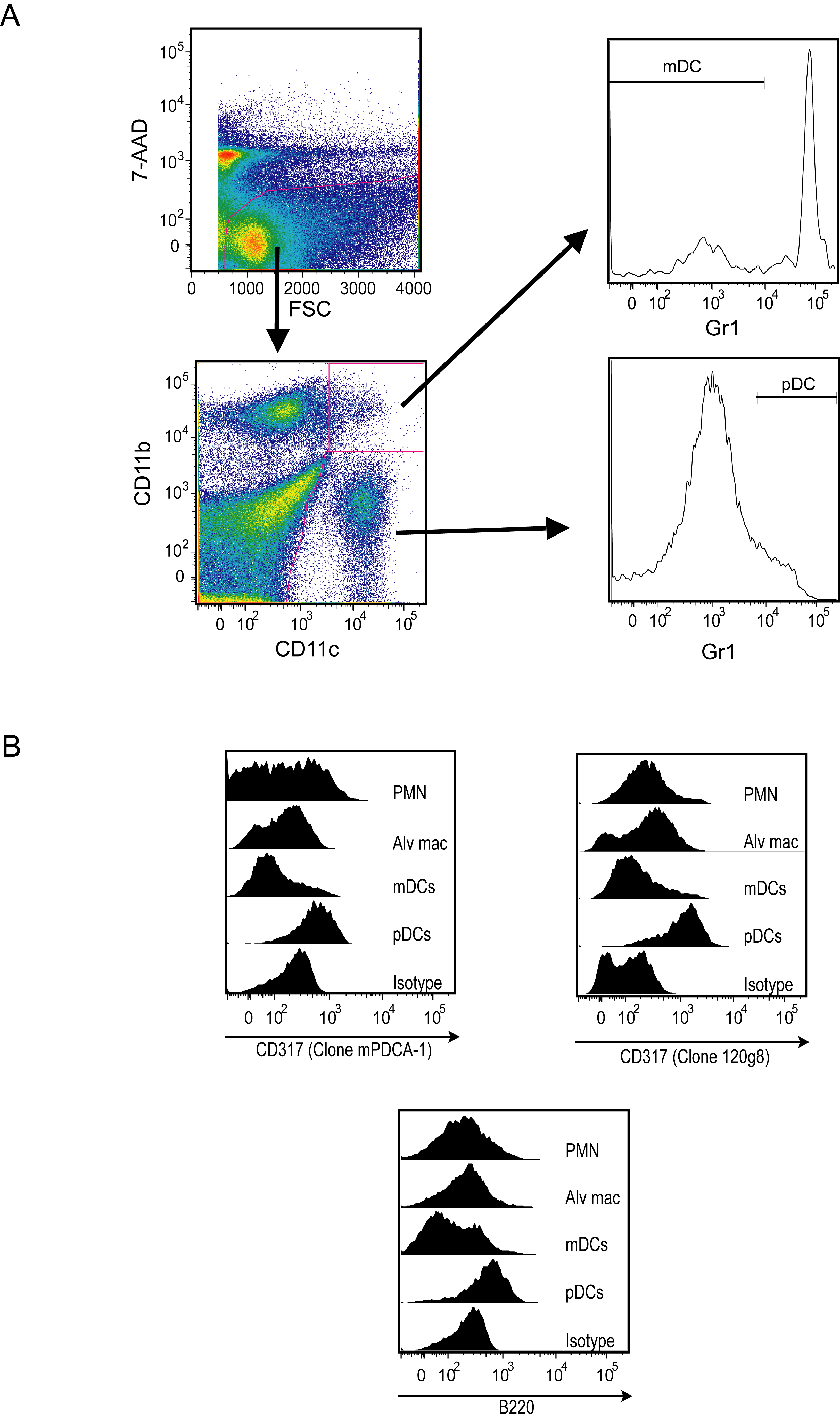

Supplement: Figure S1 — DC subsets found in the lung of mice. A/J mice treated with PBS were sacrificed, and lung cells stained with 7-AAD, and antibodies to CD11c, Gr1, and CD11b. 7-AAD- cells were gated for analysis of CD11b, CD11c, and Gr1 expression. (A) mDCs (CD11c+, CD11b+, Gr1−) and pDCs (CD11c+, CD11b−, Gr1+) cells can clearly be found in the lungs. Other major populations identified included neutrophils (CD11c+, CD11b+, Gr1+) and alveolar macrophages (CD11c+, CD11b−, Gr1−). (B) Gated DC population were analyzed for expression of B220, and CD317 with independent CD317-specific clones (120g8 and mPDCA-1). (2.49 MB TIF) [file pone.0003879.s001.tif]

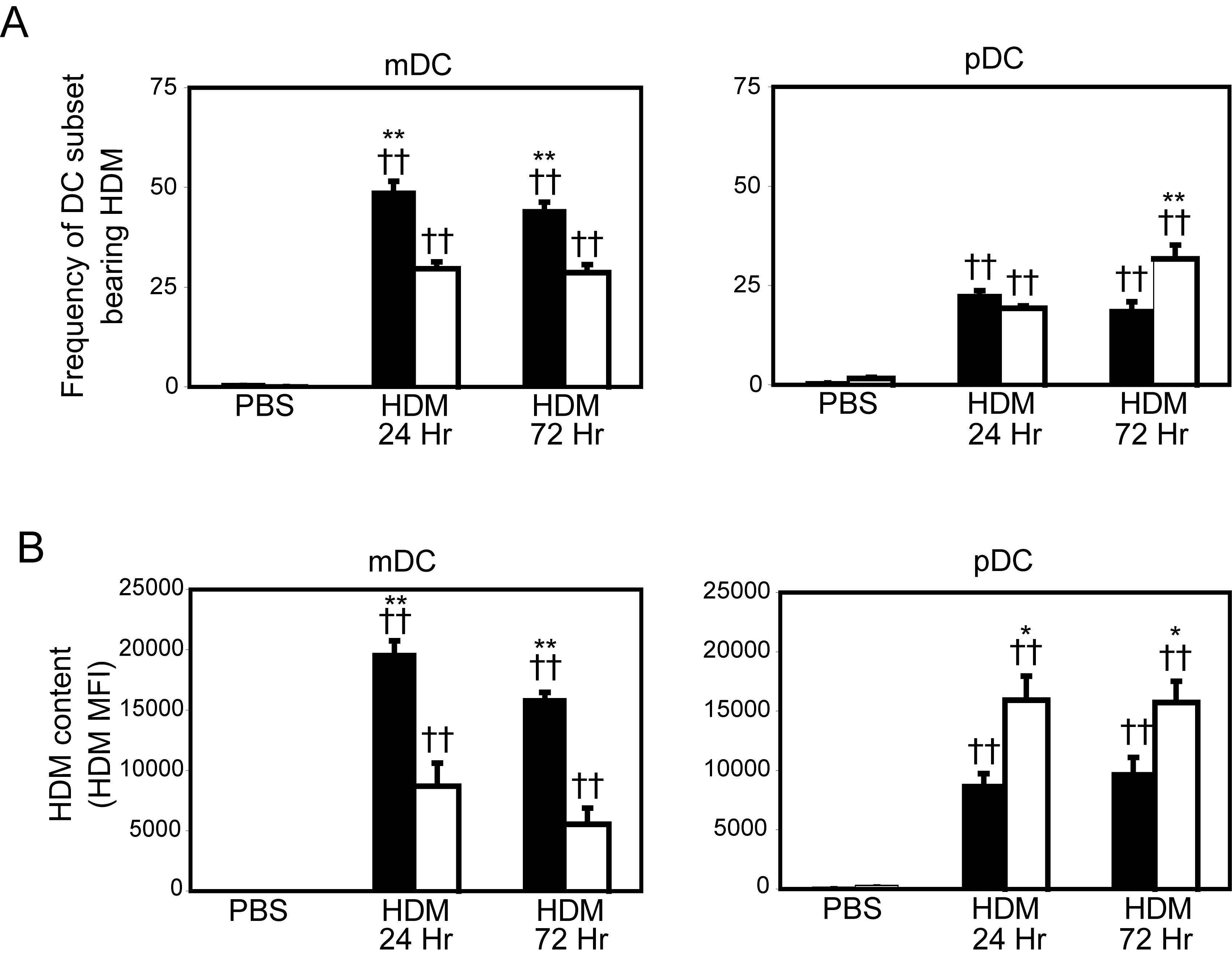

Supplement: Figure S2 — Enhanced allergen uptake by pulmonary mDCs in susceptible mice is observed in previously sensitized animals. A/J (solid bars) and C3H (open bars) mice were sensitized and subsequently challenged with PBS or AF405-HDM as described in Materials and Methods. Proportion of total mDCs (left panels) and pDCs (right panels) containing allergen (A) and allergen content (B) was determined by flow cytometry. Mean+SEM shown (n = groups of 8 mice in 2 independent experiments). * and ** indicate significant differences of p<0.05 and p<0.001 respectively between A/J and C3H animals. †† indicates significant differences compared to PBS-treated animals, p<0.001. (0.33 MB TIF) [file pone.0003879.s002.tif]

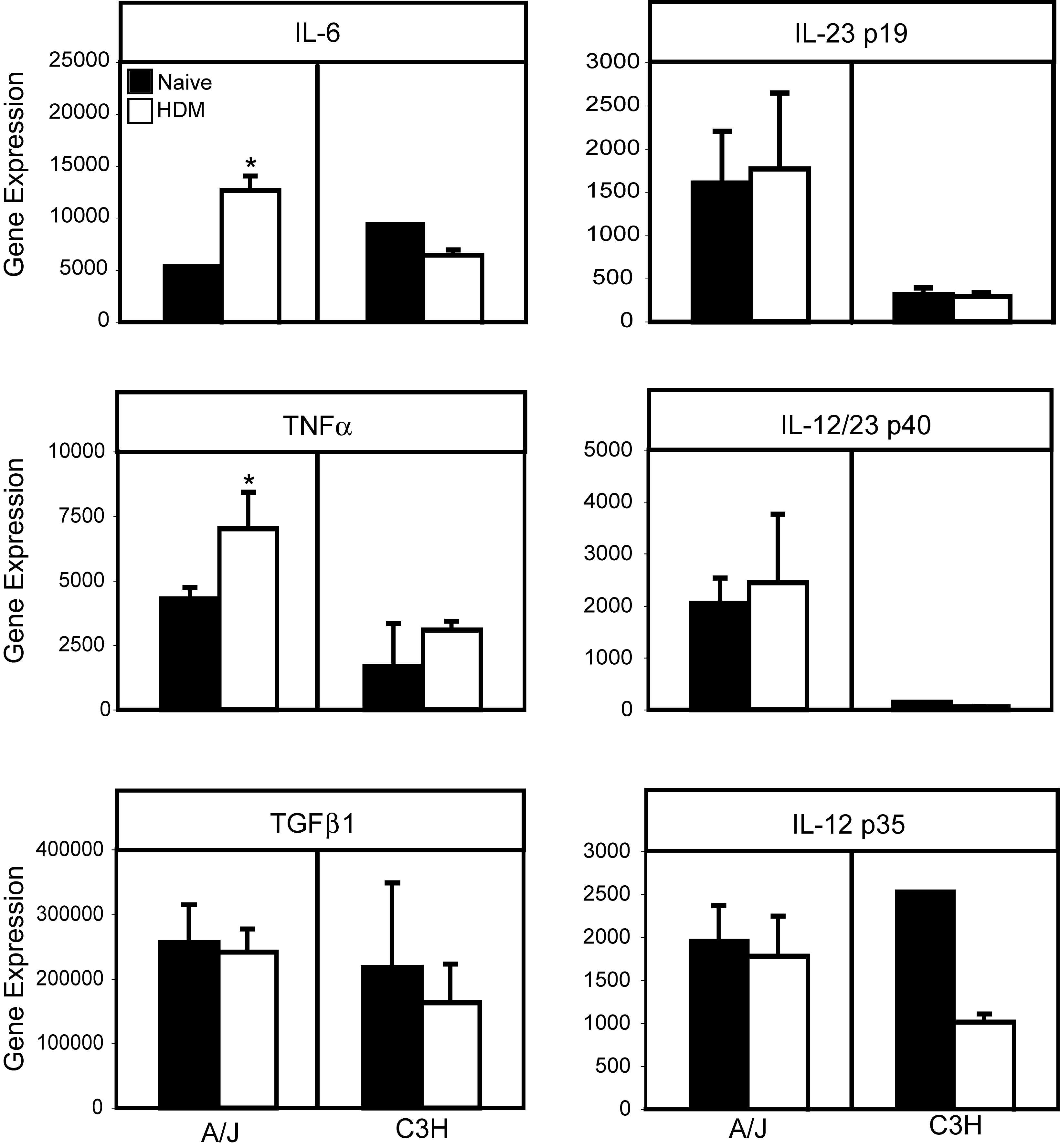

Supplement: Figure S3 — Cytokine expression in pDCs from naïve and HDM-treated mice. A/J and C3H mice were sensitized with a single dose of HDM as described in Materials and Methods. Mice were sacrificed before allergen challenge, or 48 hours after a single allergen exposure challenge, pulmonary pDCs were isolated by FACS sorting, and RNA was isolated. Expression of IL-6, IL-23 p19, TNFα, IL-12/23 p40, TGFβ1 and IL-12 p35 were determined by real time PCR. Representative data from 1 of 3 experiments shown. * indicates significant differences between A/J and C3H mice, p<0.05 (0.45 MB TIF) [file pone.0003879.s003.tif]

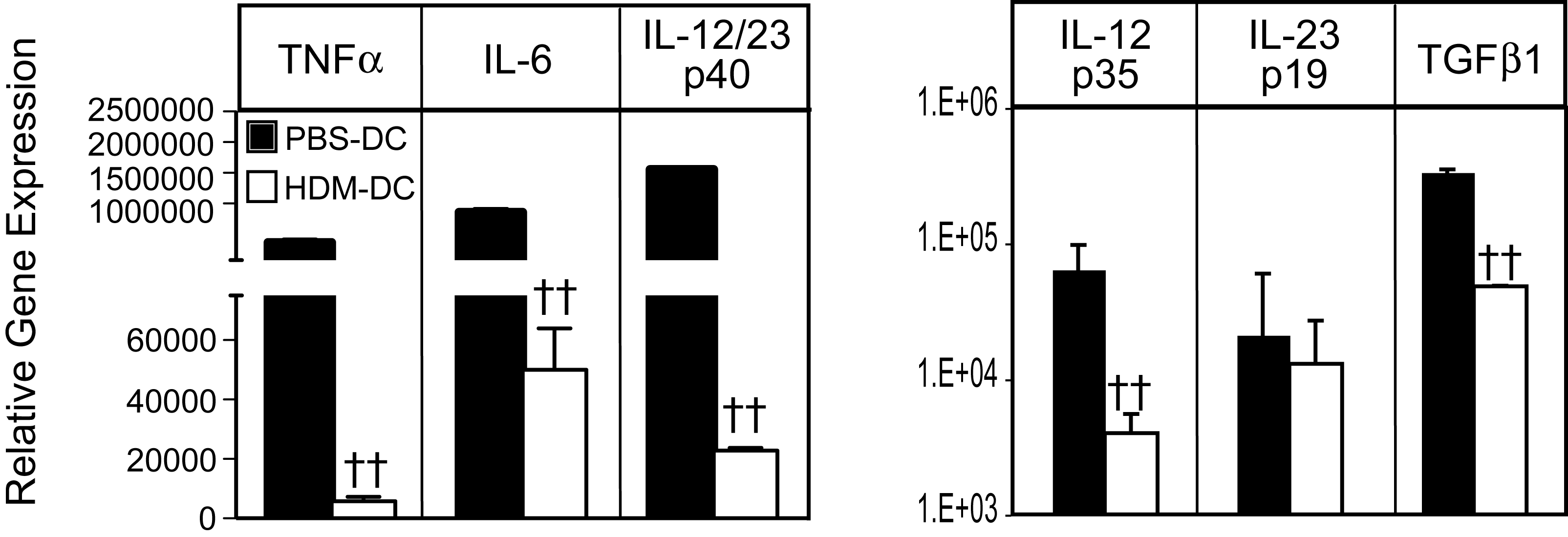

Supplement: Figure S4 — Cytokine expression by HDM-treated BMDCs from A/J mice. mDCs were derived by culturing bone marrow cells in the presence of GM-CSF and IL-4 for 6 days. On day 7, BMDC were pulsed with 30 µg/ml of HDM or medium. On day 8, BMDCs were matured by the addition of 1 µg/ml LPS. Cytokine expression by bone marrow-derived mDCs was determined by real time PCR analysis. Mean+SEM shown (n = 8 BMDC samples from 2 independent experiments). †† indicates significant differences compared to PBS-pulsed DCs, p<0.001. (0.20 MB TIF) [file pone.0003879.s004.tif]
